# Experimental recreation of the evolution of lignin-degrading enzymes from the Jurassic to date

**DOI:** 10.1186/s13068-017-0744-x

**Published:** 2017-03-16

**Authors:** Iván Ayuso-Fernández, Angel T. Martínez, Francisco J. Ruiz-Dueñas

**Affiliations:** IPSBB unit, Centro de Investigaciones Biológicas, CSIC, Ramiro de Maeztu 9, 28040 Madrid, Spain

**Keywords:** Fungal genomes, Ancestral sequence reconstruction, Fungal evolution, Lignin biodegradation resurrected enzymes, Ligninolytic peroxidases, Catalytic properties, pH stability

## Abstract

**Background:**

Floudas et al. (*Science* 336: 1715) established that lignin-degrading fungi appeared at the end of Carboniferous period associated with the production of the first ligninolytic peroxidases. Here, the subsequent evolution of these enzymes in Polyporales, where most wood-rotting fungi are included, is experimentally recreated using genomic information.

**Results:**

With this purpose, we analyzed the evolutionary pathway leading to the most efficient lignin-degrading peroxidases characterizing Polyporales species. After sequence reconstruction from 113 genes of ten sequenced genomes, the main enzyme intermediates were resurrected and characterized. Biochemical changes were analyzed together with predicted sequences and structures, to understand how these enzymes acquired the ability to degrade lignin and how this ability changed with time. The most probable first peroxidase in Polyporales would be a manganese peroxidase (Mn^3+^ oxidizing phenolic lignin) that did not change substantially until the appearance of an exposed tryptophan (oxidizing nonphenolic lignin) originating an ancestral versatile peroxidase. Later, a quick evolution, with loss of the Mn^2+^-binding site, generated the first lignin peroxidase that evolved to the extant form by improving the catalytic efficiency. Increased stability at acidic pH, which strongly increases the oxidizing power of these enzymes, was observed paralleling the appearance of the exposed catalytic tryptophan.

**Conclusions:**

We show how the change in peroxidase catalytic activities meant an evolutionary exploration for more efficient ways of lignin degradation by fungi, a key step for carbon recycling in land ecosystems. The study provides ancestral enzymes with a potential biotechnological interest for the sustainable production of fuels and chemicals in a biomass-based economy.

**Electronic supplementary material:**

The online version of this article (doi:10.1186/s13068-017-0744-x) contains supplementary material, which is available to authorized users.

## Background

The large diversity of living organisms that we observe today is associated to the evolution of proteins, making the analysis of how proteins change with time a central issue in molecular evolution. However, the study of ancestral proteins has an important difficulty: they are extinct. Therefore, the use of the tools provided by bioinformatics is mandatory [1]. To this point, ancestral sequence reconstruction can give us hints about ancient protein functions, and resurrection of the extinct proteins in the laboratory, using *ad hoc* expression hosts, will allow to directly analyze their properties and confirm evolutionary hypotheses. During the past years, several examples have shown the power of this technique, from resurrection of the most ancient proteins in the Precambrian [2–4] to the explanation of how enzymes evolved and acquired the specific mechanisms and functions that they have today [5–8].

Resurrected proteins are of interest not only because of the essential information about evolution that they can provide, but also due to the biotechnological potential that they have [9]. Ancestral proteins often have higher stability [10] and, likely, new activities [11], which can make of them interesting biocatalysts. Also, if we identify the elements that confer this stability or new activity we can improve extant enzymes by rational design. Or even more, the famous James Gould [12] sentence “replaying the tape of life” will no longer be a metaphor if we evolve the ancient proteins in the laboratory [13, 14].

A central problem of white biotechnology for establishing a sustainable bioeconomy in the twenty-first century is processing recalcitrant lignin in vascular plant feedstocks [15]. Biological decay of lignin is essential for carbon recycling in nature, and lignin removal often also represents a key step for the sustainable production of fuels and chemicals in lignocellulose biorefineries [16]. Oxidation of the predominantly nonphenolic lignin polymer is a unique ability of extracellular enzymes produced by white-rot basidiomycetes [17]. These fungi secrete three families of ligninolytic peroxidases: (i) Lignin peroxidases (LiPs), which are able to oxidize nonphenolic lignin model compounds [18]; (ii) Manganese peroxidases (MnPs), which oxidize Mn^2+^ to Mn^3+^ whose chelates act as diffusing oxidizers of phenolic lignin [19]; and (iii) Versatile peroxidases (VPs), which combine the catalytic activities (and oxidation sites) of LiPs, MnPs, and plant peroxidases acting on phenols and some dyes [20, 21]. Often white-rot basidiomycetes also produce generic peroxidases (GPs) with catalytic properties similar to the plant peroxidases [22]. These four peroxidase types, which constitute the class II of the peroxidase–catalase superfamily [23], are well characterized today and they differ in the substrate oxidation sites they have [24].

In the last years, the evolution of lignin-degrading organisms and enzymes has been investigated using genomic information. Molecular clock analyses and reconstruction of ancestral states of catalytic sites showed that the origin of lignin biodegradation occurred in the late Carboniferous with the appearance of the first ligninolytic peroxidase in the common ancestor of Agaricomycetes (Polyporales included) [25, 26]. This event provided to the first white-rot fungi the ability to attack the lignocellulosic biomass of vascular plants, enabling carbon recycling in land ecosystems. In the present work we target, by complete sequence reconstruction, the evolution that led to the highly specialized LiP of *Phanerochaete chrysosporium* (isoenzyme H8) [18, 27], the first sequenced basidiomycete as a model ligninolytic organism [28]. With this aim, we resurrected the most relevant enzymes of the evolutionary line from the common ancestor of class-II peroxidases in the order Polyporales, where most wood-rotting fungi are included [29], and studied their catalytic and stability properties with the aim of recreating in the laboratory the natural evolution of lignin-degrading enzymes, which contributed to land colonization by vascular plants [25].

## Results

### Reconstruction of ancestral sequences from Polyporales genomes

**Fig. 1:**
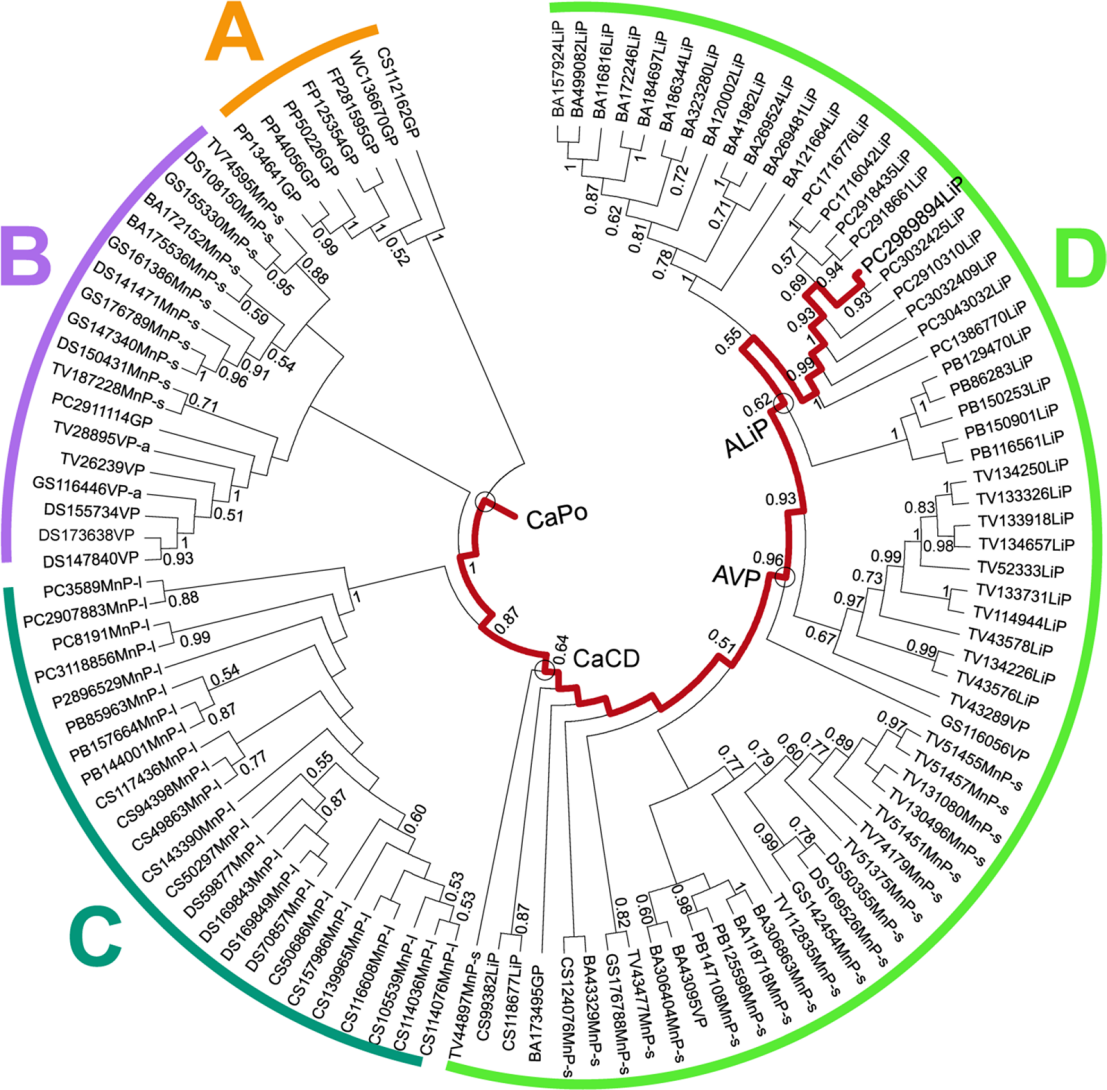
Phylogenetic tree for 113 peroxidases from ten Polyporales genomes (sequences in Additional file 2). Clusters A (GPs), B (short MnPs and VPs, plus one GP), C (long MnPs), and D (LiPs, short MnPs, and VPs, plus one GP) are shown. The path from the common ancestor to the extant LiPH8 of *P. chrysosporium* (JGI ID# 2989894) is in* red*. Also, the milestones in this evolutionary line (CaPo, CaCD, AVP, and ALiP) are marked (*circles*). The sequence labels start with the species code (BA, *Bjerkandera adusta*; CS, *Ceriporiopsis subvermispora*; DS, *Dichomitus squalens*; FP, *Fomitopsis pinicola*; GS, *Ganoderma* sp; PB, *Phlebia brevispora*; PC, *P. chrysosporium*; PP, *Postia placenta*; TV, *Trametes versicolor*; and WC, *Wolfiporia cocos*) followed by the JGI ID# and the peroxidase type, including GP, LiP, short MnP (MnP-s), long MnP (MnP-l), VP, and atypical VP (VP-a). Bootstrap values >0.5 are indicated on the different nodes

**Fig. 2:**
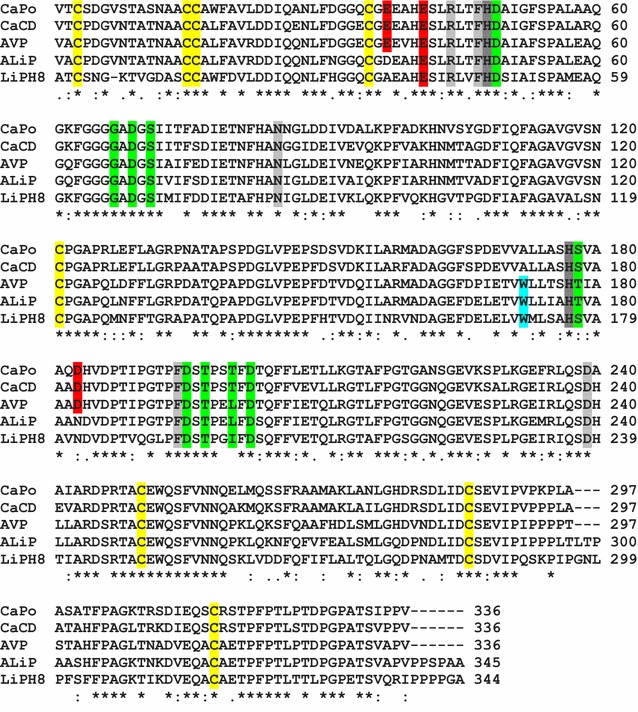
Alignment of the four most probable ancestral sequences (mature proteins) predicted with PAML 4.7 (using the WAG evolution model) and extant LiPH8 (alternative ancestral sequences are in Additional file 1: Fig. S1). Conserved catalytic and other relevant residues [24] are indicated including: two active site histidines (*dark gray*); three acidic residues forming the Mn^2+^-binding site (*red*); other active site conserved residues (*light gray*); one tryptophan involved in lignin direct oxidation (*cyan*); nine ligands of two Ca^2+^ ions (*green*); and eight cysteines forming disulfide bonds (*yellow*). Symbols below indicate full conservation of the same (*asterisk*) or equivalent residues (*colon*) and partial residue conservation (*dot*). The identity between the sequences decreased in the order: 87% (301) for AVP/ALiP, 86% (289) for CaPo/CaCD, 74% (257) for CaCD/ALiP, 74% (248) for CaPo/AVP, 72% (248) for ALiP/LiPH8, 70% (242) for CaPo/ALiP, 66% (226) for AVP/LiPH8, 61% (211) for CaCD/LiPH8, and 61% (209) for CaPo/LiPH8 (with the number of aligned residue pairs in parenthesis)

From the information available in ten genomes of Polyporales (phylum Basidiomycota) sequenced at the Joint Genome Institute (JGI), a maximum likelihood (ML) phylogenetic tree of ligninolytic peroxidase and GP sequences (Fig. 1) was constructed with RAxML [30]. The tree, which is consistent with the previous results revealing a robust evolutionary history [25, 31], was used to predict ancestral sequences using PAML 4.7 [32] and the WAG evolution model (yielding reconstructed sequences with the highest average probabilities), followed by manual curation. In the path from the first peroxidase in Polyporales to LiPH8 (Fig. 1, red line), we focused on four proteins (nodes), whose most probable reconstructed sequences are shown in Fig. 2, because they are milestones in LiP appearance: CaPo is the Common ancestor of Polyporales peroxidases, CaCD represents the Common ancestor of Cluster D (the largest peroxidase cluster including LiPs), AVP would be the most Ancestral VP in this evolutionary line, and ALiP would be the most Ancestral LiP in Polyporales (and probably in all basidiomycetes).

The posterior probabilities for three of the most probable ancestral sequences (CaCD, AVP, and ALiP) are high (mean ≥ 0.95) while those of the first ancestor (CaPo) were lower (mean 0.82) (Additional file 1: Fig. S2). To check the possible functional variability of the four reconstructed nodes, a total of 5000 sequences (including the most probable ones) were obtained for each of them by Monte Carlo sampling [4] from the PAML results, and manually inspected for the presence/absence of the two substrate oxidation sites described below (Glu37/Glu41/Asp183 binding Mn^2+^, and Trp172 oxidizing nonphenolic lignin). For the three more recent nodes 100% of the sequences showed the following invariable oxidation site/s: Trp172 in ALiP, both Trp172 and Glu37/Glu41/Asp183 in AVP, and Glu37/Glu41/Asp183 in CaCD sequences. Therefore, only the most probable sequences were resurrected for each of them.

For node CaPo, all sequences lack Trp172 and have at least two of the above acidic residues, a situation associated to Mn^2+^ oxidation ability by normal or atypical MnPs (lacking one of these residues) [25, 31]. To verify the activity of the predicted atypical MnPs, the corresponding sequences were classified into two subsets (with either Asp37 or Arg183) that were submitted to a random sampling. In this way, the CaPo-bis and CaPo-tris alternative sequences (“near-ancestors”) were selected, which were resurrected together with the most probable CaPo ancestor with a typical Mn^2+^-oxidation site. Although the resulting sequences were not resurrected, parallel random samplings were also performed on the CaCD, AVP, and ALiP sets for in silico analysis, showing invariable catalytic sites (their sequences and posterior probability values are shown in Additional file 1: Figs. S1 and S2, respectively, together with those of the CaPo node).

### In silico analysis of ancestral heme pocket and substrate oxidation sites

Molecular models of the selected ancestral proteins, together with multiple alignments, reveal that their 12 helices, two sites binding structural Ca^2+^ ions, and four disulfide bonds did not change during evolution, despite the differences in sequence (only 61–87% identity) between the ancestors and between them and the extant LiPH8 (see Fig. 2 legend). This comparison also reveals that most of the essential amino acids for LiPH8 function were already present at the first stages of evolution, including proximal His177 (near the heme iron), Asp239, and Phe194 at one side of heme, and distal His48, Arg44 (both contributing to H_2_O_2_ reaction in extant peroxidases), Asn85, and Phe47 at the opposite side. Hypothetical His177-Asp239 and Ser/Thr178-Asp202 H-bonds would control the position of the proximal histidine with respect to the heme iron (Additional file 1: Fig. S3). The proximal Ca^2+^ hypothetical ligands—Ser178 (CaPo and CaCD) or Thr178 (AVP and ALiP), Asp195, Thr197, Thr200 (CaPo, CaCD), or Leu200 (AVP and ALiP), and Asp202—show some differences with those in LiPH8 (Ser177, Asp194, Thr196, Ile199, and Asp201), while the distal Ca^2+^ ligands would be exactly the same. However, the above differences would not affect proximal Ca^2+^ binding since backbone carbonyls are involved at those positions where differences are predicted.

**Fig. 3:**
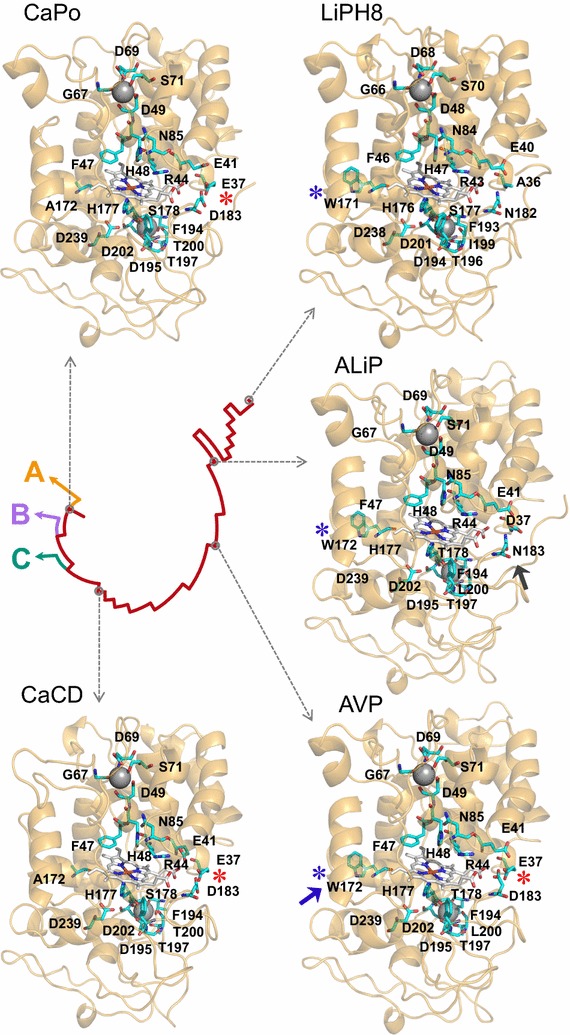
Molecular models of the reconstructed ancestors and extant LiP. The hypothetical location of heme, two Ca^2+^ ions, ancestral Mn^2+^-binding site formed by two glutamate and one aspartate residues (*red asterisks*), evolved lignin-oxidizing exposed tryptophan (*blue asterisks*), and other relevant residues (Fig. 2) are shown on the ancestor models, built using three related crystal structures (4BM1, 3FJW, and 1QPA) as templates [57], and LiPH8 crystal structure (PDB 1B82). The models are shown on a schematic representation of evolution (see Fig. 1). The *blue* and *gray arrows* show the amino acids that appeared in the main evolutionary events and defined new activities (gain of catalytic Trp172 and loss of Mn^2+^-binding site, respectively)

Then, several substrate oxidation sites were identified in the molecular models and sequences of the ancestral peroxidases. The three acidic residues that define the Mn^2+^-binding site (red background and asterisks in Figs. 2 and 3, respectively) of extant MnP [33] and VP [34] already appear in CaPo (Glu37, Glu41, Asp183), and continue invariably in evolution until the appearance of ALiP, when Glu37 becomes Asp37 and Asp183 changes to Asn183 (gray arrow in Fig. 3). Extant LiPs and VPs oxidize nonphenolic (high redox-potential) lignin model compounds by long-range electron transfer from an exposed tryptophan, being Trp171 in LiPH8 and Trp164 in *Pleurotus eryngii* VP [35–37], whose implication in direct oxidation of nonphenolic lignin has been recently demonstrated [38]. Analysis of the corresponding residues revealed that CaPo and CaCD have an alanine (Ala172) in that position. The fact that these first two (most probable) ancestors have a Mn^2+^-binding site, but no catalytic tryptophan, makes of them two putative MnPs (the catalytic activity of the CaPo alternative sequences was established after heterologous expression described below). Then, we were able to track the point in the evolutionary line where the catalytic tryptophan (blue asterisk in Fig. 3) appears: in AVP Ala172 becomes Trp172 (Fig. 3 blue arrow). Since AVP has also a well-structured Mn^2+^-binding site, this protein is a priori a VP. Finally, in ALiP the Trp172 is conserved but the Mn^2+^-binding site disappears becoming the first LiP in the evolution of Polyporales. To confirm the above predictions, the most relevant sequences were resurrected.

### Kinetic properties of resurrected peroxidases

The most probable ancestral and LiPH8 DNA sequences (plus two additional sequences from node CaPo) were synthesized, expressed in *Escherichia coli*, in vitro activated, and purified to homogeneity. Then, their steady-state kinetic constants for the oxidation of veratryl alcohol (VA) representing nonphenolic lignin, 2,6-dimethoxyphenol (DMP) representing the minor phenolic moiety in lignin, and Mn^2+^ were estimated, together with those for two dyes with high (Reactive Black 5, RB5) and low (2,2′-azinobis[3-ethylbenzothiazoline-6-sulfonate], ABTS) redox-potentials (Table 1).

**Table 1:** Kinetic parameters—*K*_m_ (µM), *k*_cat_ (s^−1^), and *k*_cat_ */K*_m_ (s^−1^ mM^−1^)—for the oxidation of Mn^2+^, phenolic (DMP) and nonphenolic (VA) aromatics, and dyes (means and 95% confidence limits) by the resurrected enzymes and extant LiPH8 (a comparison of all catalytic efficiencies is shown in Fig. 4)

	CaPo	CaCD	AVP	ALiP	LiPH8
Metal ion					
Mn^2+^					
* K*_m_	700 ± 48	275 ± 41	62 ± 10	–^a^	–
* k*_cat_	185 ± 3	170 ± 6	106 ± 4	–	–
* k*_cat_ */K*_m_	260 ± 15	617 ± 80	1710 ± 240	–	–
Aromatics					
DMP^b^ (low efficiency)					
* K*_m_	32,900 ± 2700	66,800 ± 3800	32,500 ± 12,100	–	–
* k*_cat_	221 ± 9	109 ± 4	31 ± 5	–	–
* k*_cat_ */K*_m_	6.7 ± 0.3	1.6 ± 0.03	1.0 ± 0.2	–	–
DMP (high efficiency)					
* K*_m_	–	–	5.3 ± 1.1	34.0 ± 5.4	4.0 ± 0.07
* k*_cat_	–	–	4.5 ± 0.1	18.3 ± 0.7	6.9 ± 0.5
* k*_cat_ */K*_m_	–	–	837 ± 162	537 ± 55	600 ± 36
VA					
* K*_m_	–	–	299 ± 104	773 ± 155	79.3 ± 18
* k*_cat_	–	–	7.1 ± 0.5	21.3 ± 1.0	16.2 ± 0.8
* k*_cat_ */K*_m_	–	–	24 ± 7	28 ± 5	205 ± 4
Dyes					
ABTS^b^ (low efficiency)					
* K*_m_	3170 ± 270	1280 ± 350	2150 ± 420	–	–
* k*_cat_	539 ± 24	103 ± 10	25 ± 2	–	–
* k*_cat_ */K*_m_	170 ± 8	80 ± 15	12 ± 1	–	–
ABTS (high efficiency)					
* K*_m_	–	–	5.4 ± 0.7	13.7 ± 3.5	21.3 ± 2.3
* k*_cat_	–	–	2.1 ± 0.1	12.5 ± 0.7	6.5 ± 0.2
* k*_cat_ */K*_m_	–	–	400 ± 44	911 ± 212	300 ± 25
RB5					
* K*_m_	–	–	4.8 ± 0.8	12.6 ± 3.6	–
* k*_cat_	–	–	2.4 ± 0.2	5.4 ± 0.8	–
* k*_cat_ */K*_m_	–	–	504 ± 50	428 ± 68	–
Direction of evolution					

The alternative CaPo ancestors oxidized DMP (low efficiency) with *K*_m_ 455,000 ± 84,000 (CaPo-bis) and 298,000 ± 66,000 μM (CaPo-tris), *k*_cat_ 129 ± 22 (CaPo-bis) and 108 ± 21 s^−1^(CaPo-tris), and *k*_cat_ */K*_m_ 0.3 ± 0.05 (CaPo-bis) and 0.4 ± 0.01 s^−1^mM^−1^ (CaPo-tris); ABTS (low efficiency) with *K*_m_, 1392 ± 345 (CaPo-bis) and 807 ± 104 μM (CaPo-tris), *k*_cat_, 241 ± 23 (CaPo-bis) and 128 ± 6 s^−1^ (CaPo-tris), and *k*_cat_ */K*_m_, 173 ± 28 (CaPo-bis) and 160 ± 15 s^−1 ^ mM^−1^ (CaPo-tris), while Mn^2+^ was only oxidized by CaPo-tris with *K*_m_ 5010 ± 274 µM, *k*_cat_ 47 ± 1 s^−1^, and *k*_cat_ */K*_m_ 9.3 ± 0.4 s^−1^ mM^−1^

Reactions at 25 °C in 0.1 M tartrate at optimal pH 5.0 (CaPo, and its alternative ancestors, and CaCD) or 5.5 (AVP) for Mn^2+^, pH 3.5 (AVP) or 3.0 (ALiP and LiPH8) for VA, pH 2 (CaPo) or 3.0 (others) for DMP, pH 3.0 (CaPo) and pH 3.5 (others) for ABTS, and pH 3 for RB5; and saturating H_2_O_2_ concentrations of 0.4 mM for CaPo, CaCD, and AVP, 0.2 mM for ALiP, and 0.1 mM for LiPH8

^a^–Absence of activity

^b^Biphasic kinetics for DMP and ABTS oxidation by AVP enabled calculation of two sets of constants assigned to two catalytic sites, as reported for extant VP [40], comparable to those present in CaPo/CaCD and ALiP/LiPH8, respectively

The above substrates are oxidized at different sites, and the kinetic analysis shows how these sites have evolved giving rise to different peroxidase families. The sites for oxidation of Mn^2+^ and high redox-potential substrates such as VA (and RB5) have been already described above. Phenols, such as DMP (and the generic oxidoreductase substrate ABTS) can be oxidized: (i) with high efficiency at the same tryptophan oxidizing the high redox-potential substrates; and (ii) with low efficiency at one of the heme access channels [39, 40].

**Fig. 4:**
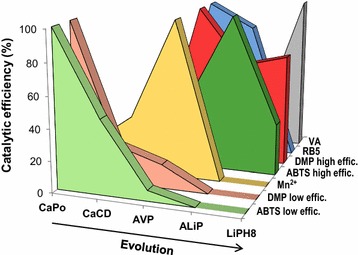
Catalytic efficiency on different substrates during peroxidase evolution. Changes of the relative catalytic efficiencies (*k*_cat_ */K*_m_) on ABTS, DMP, Mn^2+^, RB5, and VA (the two former at high and low efficiency oxidation sites) during evolution, with the maximum for each substrate taken as 100%. The CaPo-bis and CaPo-tris alternative ancestors showed the following relative catalytic efficiencies: (i) ABTS (low efficiency), 100 and 94%, respectively; (ii) DMP (low efficiency), 4.5 and 6.0%, respectively; (iii) Mn^2+^, 0.5% for CaPo-tris (no activity detected with CaPo-bis); and (iv) no activity on ABTS (high efficiency), DMP (high efficiency), RB5, and VA

The catalytic efficiency (*k*_cat_/*K*_m_) on the different substrates clearly showed the changes produced along the evolution, as illustrated in Fig. 4. In this way, the ability to oxidize Mn^2+^ gradually improved from CaPo to AVP (due to 11-fold *K*_m_ reduction) and then was completely lost in ALiP. In contrast, CaPo shows the highest activity oxidizing low redox-potential DMP (and ABTS) at the low efficiency site (heme channel), which is reduced from CaPo to AVP (7–15 fold lower catalytic efficiency, due to *k*_cat_ decrease) and then disappears. The two alternative CaPo ancestors showed similar activity on ABTS and lower or null on DMP and Mn^2+^ (Table 1 footnote), and were classified as GP (CaPo-bis) and atypical MnP (CaPo-tris). The vanishing of the low efficiency oxidation site comes along with the rise of a high efficiency site in AVP. In this ancestral peroxidase, DMP (and ABTS) begins to be oxidized at a second site in the protein (the catalytic tryptophan discussed below), as revealed by sigmoid kinetic curves.

As regard for the high redox-potential substrates, the turning point where the LiP/VP usual substrate VA (and the recalcitrant dye RB5) begins to be oxidized in this evolutionary line is AVP, with the appearance of the exposed Trp172 at a position that coincides with the catalytic tryptophan in extant VP and LiP [35–37]. The catalytic efficiency towards VA is maintained from AVP to ALiP and then sharply increases (sevenfold) from ALiP to LiPH8. The *k*_cat_ value did not change, and the higher catalytic efficiency is due to an improvement in VA affinity (tenfold lower *K*_m_ value). The appearance of Trp172 also resulted in efficient oxidation of the low redox-potential substrates, fully substituting the low efficiency site in ALiP and extant LiP. The activity of the catalytic tryptophan in the advanced stages of peroxidase evolution will be affected by the electrostatic charge of its surface environment (Additional file 1: Fig. S4): VA cation radical will be stabilized by the strongly acidic environment in LiPH8 that, in contrast, will prevent the oxidation of anionic RB5.

### Stability of ancestral peroxidases

**Fig. 5:**
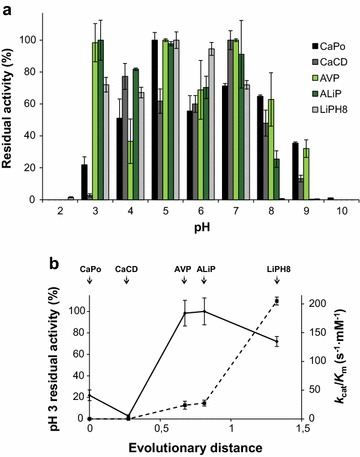
pH stability of the resurrected ancestors and extant LiPH8. **a** Residual activities were measured with 2.5 mM ABTS at optimal pH (see Table 1) after 4 h incubation at different pH values (25 °C) and referred to activity after 1 min incubation of each of them at pH 5. The CaPo-bis and CaPo-tris alternative ancestors showed the following relative residual activities: (i) pH 2, 0%; (ii) pH 3, 10 and 44%, respectively; (iii) pH 4, 88 and 89%, respectively; (iv) pH 5, 85 and 90%, respectively; (v) pH 6, 93 and 97%, respectively; (vi) pH 7, 93 and 95%, respectively; (vii) pH 8, 84 and 95%, respectively; (viii) pH 9, 88 and 28%, respectively; and (ix) pH 10, 0%. **b** Comparison of the VA catalytic efficiency (s^−1^ mM^−1^; *dashed line*) and enzyme stability at pH 3 (*continuous line*) vs evolutionary distance [31]. The pH 3 residual activities were measured as indicated in **a**. Means and 95% confidence limits

The pH stability of the resurrected peroxidases was analyzed by measuring the residual activity (after 4 h at 25 °C) in the pH 2–10 range (Fig. 5a). All the enzymes retained ≥50% activity at pH 5–7, which strongly decreased at pH 8–10. The most important difference was at pH 3, where AVP, ALiP, and LiPH8 retained ≥70% activity, while CaPo (alternative ancestors included) and CaCD were strongly inactivated. As shown in Fig. 5b, the increase of acidic pH stability paralleled the introduction of VA oxidation activity (due to the appearance of catalytic Trp172) and was maintained till today.

**Fig. 6:**
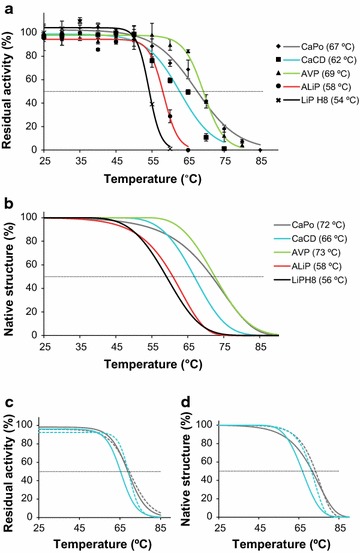
Changes in thermal stability. **a**, **b** Thermal stability of the resurrected proteins and LiPH8 estimated from enzyme activity (**a**) and secondary structure loss (**b**). The *T*_50_ and *T*_*m*_ values are provided in the legends. Alternative CaPo-bis and CaPo-tris showed 67 and 63 °C *T*_50_ and 69 and 66 °C *T*_*m*_ values, respectively. **c**, **d** Mn^2+^ (1 mM) addition during incubation (*dashed lines*) slightly increased the *T*_50_ (**c**) and *T*_m_(**d**) values of the CaPo (*gray*) and CaCD (*blue*) ancestors. Inactivation was measured after 10 min at pH 5.5, using 2.5 mM ABTS (**a**) or 6 mM Mn^2+^ (**c**) as substrate. Secondary structure loss (**b**, **d**) was estimated by circular dichroism at 222 nm

Thermal stability was analyzed by both residual activity measurements and thermal melting profiles from circular dichroism (Fig. 6a, b respectively). From most probable CaPo (and alternative ancestors) to LiPH8 there is a decrease in thermal stability, but AVP leaves that trend. Moreover, Mn^2+^ caused a slight increase of the thermal stability, as revealed by *T*_50_/*T*_*m*_ values (Fig. 6c, d), as reported for other MnPs [39].

## Discussion

Over the last years there has been a profound mining of basidiomycete genomes to find new enzymes and obtain evolutionary information [25, 31, 41, 42]. Our analysis of the lineage from the most ancestral peroxidase in Polyporales, a basidiomycete order that appeared near 150 mya at the end of the Jurassic [25, 26], to *P. chrysosporium* LiP [18, 27] reveals how these enzymes acquired the ability to degrade lignin and how this ability changed with time. At the same time, the reconstruction of ancestral peroxidases provides proteins of biotechnological interest in lignocellulose biorefineries [16] (e.g., CaPo is a thermostable enzyme and a good candidate for further directed evolution, as it has the potential to become every peroxidase in the phylogenetic tree).

In the above analysis, we used PAML [32], a common software for ancestral protein reconstruction, and the published ML phylogenetic tree of class-II peroxidases in Polyporales [31], whose accuracy is assured by a precise annotation of the peroxidase genes. The program uses the best evolutionary model for the sequences analyzed (WAG model in this case) to predict the most probable amino acid at every position of each node in the phylogenetic tree (providing a complete matrix with the probabilities of every other amino acids). The reconstruction procedure has inherent limitations for sequence prediction in the most ancestral nodes (such as CaPo) that can be partially overcome by sequence sampling from the PAML matrix and in silico and/or experimental evaluation of ancestor variability (reconstruction robustness) with respect to the property/ies most relevant for each phylogenetic analysis (catalytic properties defining the different peroxidase types, in the present study).

Ligninolytic peroxidases are high redox-potential enzymes being this characteristic related to the distance between the heme iron and the proximal histidine (Nε) acting as its fifth ligand [43, 44]. This distance is shorter in the peroxidases of prokaryotic origin, such as cytochrome *c* peroxidase (C*c*P), and significantly increased in fungal peroxidases. This is due to an H-bond between a backbone carbonyl (LiPH8 Asp201) and the hydroxyl of a Ser/Thr residue (LiPH8 Ser177, absent from C*c*P) that displaces the contiguous proximal histidine increasing the electron deficiency of iron in ligninolytic and other eukaryotic peroxidases. The distance between the His177Nε and the heme iron in the molecular models of the ancestral peroxidases are not significantly different from those found in LiP, due to conserved Ser/Thr178-Asp202 and His177-Asp239 H-bonds, although significantly larger than in C*c*P (Additional file 1: Fig. S3). Therefore, no strong changes in peroxidase redox-potential are expected to be produced during LiP evolution in Polyporales, and the VA oxidation ability of AVP (and ALiP) would be mainly related to the appearance of the surface catalytic tryptophan.

It is important that the heme peroxidase redox-potential, and lignin-degrading ability of basidiomycete peroxidases, drastically increases when pH decreases [45]. Therefore, the improved stability at acidic pH in the most recent stages of peroxidase evolution in Polyporales (from AVP to extant LiP) represents an important evolutionary adaptation to the acidic conditions where ligninolysis occurs in nature [46]. Concerning thermal stability, AVP appears as the most thermostable ancestral peroxidase in Polyporales, especially when compared with its descendants. Experimental evidence is required to know if genetic drift [47, 48] caused the increased stability of AVP related with the appearance and subsequent evolution of the catalytic tryptophan. After that, the loss of the Mn^2+^-binding site caused a decrease in stability, since the Mn-binding site can (in the presence of Mn^2+^ or other cations) contribute to anchor the heme cofactor [19, 34], but proteins were still stable in order to be selected in evolution. In agreement with the present results, increased thermal stability has been reported for both an ancestral plant peroxidase [49] and a LiP containing several ancestral mutations [50].

The kinetic constants of the resurrected peroxidases show the high evolvability of these enzymes incorporating new activities. Although some exceptions have been reported [51, 52], it is generally assumed that ancestral enzymes have a wide substrate specificity and are specialized after duplication events [4, 6, 53]. However, peroxidases in Polyporales show a different evolutionary history. The first ancestor (CaPo) is able to oxidize only low redox-potential substrates, and Mn^2+^ with low catalytic efficiency. This low Mn^2+^-oxidizing activity is in agreement with the GP-type ancestor of Dikarya peroxidases [25]. Similar substrate specificity has been reported for the extant short MnPs, where CaPo and CaCD would be included according to their short C-terminal tail [39]. This suggests that short MnPs are old enzymes, whose efficiency oxidizing Mn^2+^ was improved later. Then, the efficiency oxidizing Mn^2+^ increased in AVP (the first VP in Polyporales) with activity on this cation similar to extant VP [34]. What is surprising, as mentioned above, is that the AVP appearance, a relatively recent event in the mid-term evolution of Polyporales peroxidases, resulted in the enzyme with the widest specificity: AVP is able to oxidize every substrate assayed. Then, evolution focused in the direction of more efficient oxidation of nonphenolic lignin at Trp172, as shown using VA, and resulted in the appearance of LiP, the most specialized ligninolytic enzyme only reported in the order Polyporales [25, 31, 42]. However, this “unusual” evolutionary behavior is most probably related to the existence of three different substrate oxidation sites in these peroxidases, whose appearance/disappearance caused qualitative changes in catalytic activities (compared with enzymes with a progressively specialized unique catalytic site).

From an organismal point of view, the above evolutionary trend can be seen as a search for new and more efficient tools to degrade lignin. From their appearance in the late Jurassic, Polyporales had ligninolytic peroxidases, as shown by the ancestral enzyme reconstruction. The oldest ancestors would use Mn^2+^, being Mn^3+^ able to oxidize the minor phenolic moiety of lignin [16]. Trp172 appeared later giving versatile AVP that oxidizes Mn^2+^ (more efficiently than its MnP-type ancestors), phenols, and most probably also nonphenolic lignin, as shown by its ability to oxidize high redox-potential substrates. When ALiP appeared, the Mn^2+^-binding site was lost, as well as the low efficiency site oxidizing phenols. In this way, non-competitive inhibition of lignin oxidation was prevented. Although the ability to oxidize Mn^2+^ was lost in the evolutionary line leading to LiPH8, a family of highly efficient Mn^2+^-oxidizing peroxidases (cluster C) evolved from the first peroxidase ancestor CaPo. Finally, after the appearance of the exposed tryptophan in AVP, the peroxidase catalytic efficiency oxidizing VA was improved, first by increasing the *k*_cat_ in ALiP and later by improving the *K*_m_ in LiPH8, which means a peroxidase specialization towards lignin degradation.

## Conclusions

We predict that the evolutionary pathway leading to the most efficient lignin-degrading enzymes (LiP family, only found in Polyporales) included successive incorporation of: (i) a surface tryptophan to an ancestral short MnP resulting in an ancestral VP; and (ii) loss of the Mn^2+^-binding site generating the first LiP. The experimental evaluation of catalytic properties of the resurrected ancestral enzymes was consistent with the bioinformatic analysis and prove the above hypothesis. Interestingly, an increase of stability at acidic pH was found simultaneously with the appearance of the catalytic tryptophan, enabling these enzymes to act under the acidic conditions characterizing lignin decay. The evolutionary and experimental studies also show that some ancestral peroxidases are of biotechnological interest because of their stability and potential evolvability.

## Methods

### Phylogenetic analysis

113 predicted peroxidase sequences (mature proteins) from the genomes of ten Polyporales (phylum Basidiomycota) species (namely *B. adusta*, *C. subvermispora*, *D. squalens*, *F. pinicola*, *Ganoderma sp*., *P. brevispora*, *P. chrysosporium*, *P. placenta*, *T. versicolor*, and *W. cocos*) available at the DOE JGI Mycocosm portal (as http://genome.jgi.doe.gov/Bjead1_1/Bjead1_1.home.html, http://genome.jgi.doe.gov/Cersu1/Cersu1.home.html, http://genome.jgi.doe.gov/Dicsq1/Dicsq1.home.html, http://genome.jgi.doe.gov/Fompi3/Fompi3.home.html, http://genome.jgi.doe.gov/Gansp1/Gansp1.home.html, http://genome.jgi.doe.gov/Phlbr1/Phlbr1.home.html, http://genome.jgi.doe.gov/Phchr2/Phchr2.home.html, http://genome.jgi.doe.gov/Pospl1/Pospl1.home.html, http://genome.jgi.doe.gov/Trave1/Trave1.home.html and http://genome.jgi.doe.gov/Wolco1/Wolco1.home.html, respectively), all of them containing one or several peroxidases of the peroxidase–catalase superfamily [31], have been used in this study (Additional file 2 **)**.

The amino-acid sequences were aligned with MUSCLE as implemented in MEGA 7 [54]. ML analysis was then performed using RAxML [30] under the GTR model with GAMMA-distributed rate of heterogeneity, using the WAG evolution model, as suggested by ProtTest [55].

### Ancestral sequence reconstruction

PAML 4.7 package [32] was used to obtain the posterior amino-acid probability per site in each ancestor under the WAG model of evolution, and the most probable whole sequences for each of the nodes, using as inputs the ML phylogeny and the MUSCLE alignment previously obtained (PAML reconstructions using the LG and Dayhoff evolution models were also performed for comparison). Marginal reconstruction was selected for the present work.

Five thousand sequences (including the most probable ancestor predicted by PAML) were selected for each of the nodes by successive Monte Carlo sampling steps (using an *ad hoc* program kindly provided by Dr J.M. Sanchez-Ruiz and 0.2 and 0.5 probability thresholds, referred to the highest probability at every position) [4]. These sequences were manually corrected for C-terminal and other insertions or deletions (the former often originating from intron to exon transitions) according to the sequences of the ancestor progeny, and inspected for the presence/absence of the substrate oxidation sites (Glu37, Glu40, and Asp183 involved in Mn^2+^ binding, and Trp172 responsible for VA oxidation).

The 5000 CaPo sequences were classified into three subsets corresponding to sequences containing: (i) the three acidic residues forming a typical Mn^2+^-binding site (including the most probable ancestor); (ii) an atypical Mn^2+^-binding site formed by only two of the above acidic residues, without a basic residue in the third position (including CaPo-tris selected later); and (iii) a site formed by two of the above acidic residues plus a basic residue in the third position (including CaPo-bis selected later), with Trp172 being absent in all the cases. The Monte Carlo sampling provided a significant number of alternative sequences with an arginine (or lysine) residue in position 183 (forming the above subset-iii) whose presence prevented binding and oxidation of the Mn^2+^ cation (as confirmed after CaPo-bis resurrection). However, for the most probable sequence, PAML reconstructed an aspartic acid at this position (resulting in a functional Mn^2+^-binding site after CaPo resurrection) in agreement with the extant peroxidases from the ten Polyporales genomes analyzed (with only 5% sequences containing a basic residue at this position).

Each of the CaPo subsets was submitted to a random sampling, together with the whole sets for the three other nodes (where no subsets were defined because the 5000 sequences from each of them showed the same catalytic sites), yielding 12 representative sequences, including the four most probable ones, that were analyzed in silico.

Then, the DNA sequences encoding the most probable amino-acid sequences for the four reconstructed nodes, together with the two alternative sequences from the CaPo subsets, were synthesized by ATG:biosynthetics (Merzhausen, Germany) after optimizing the codon usage for high expression in *E. coli* using OPTIMIZER [56].

### Protein modeling

Molecular models of the predicted proteins were obtained at the Swiss-Model automated protein homology modeling server [57] using related crystal structures, selected using the GMQE parameter, as templates (PDB entries 4BM1, 3FJW, and 1QPA corresponding to *Pleurotus ostreatus* MnP, *P. eryngii* VP, and *P. chrysosporium* LiP, respectively). All protein models had great quality taking into account the Swiss-Model parameters (good QMEAN and high GMQE). The electrostatic surfaces were computed with the PyMOL Molecular Graphics System, version 1.8 Schrödinger, LLC (http://pymol.org) using default parameters.

### *E. coli* expression

After gene synthesis, the coding sequences of the most probable CaPo, CaCD, AVP, and ALiP ancestral sequences, plus the two additional CaPo sequences described above (CaPo-bis and CaPo-tris), and the extant LiPH8 were cloned into the expression vector pET23b(+) (Novagen). The resulting plasmids were transformed into *E. coli* DH5α for propagation and conservation, and into BL21(DE3)pLysS (ancestors) or W3110 (LiPH8) for expression. With this purpose, cells were grown in Terrific broth until an OD_600_ 0.5 to 0.6 to reach an adequate expression level, induced with 1 mM isopropyl-β-d-thiogalactopyranoside, and grown for another 4 h.

The apoenzymes accumulated in inclusion bodies, as reveled by sodium dodecyl sulfate–polyacrylamide gel electrophoresis, and were activated in vitro [41, 58]. After solubilization in 8 M urea, the refolding conditions for the ancestral proteins included: 0.16 M urea, 5 mM CaCl_2_, 15 µM hemin, 0.4 mM oxidized glutathione, 0.1 mM dithiothreitol, and 0.1 mg/mL of protein in 50 mM Tris–HCl, pH 9.5. The refolding conditions for LiPH8 were: 2.1 M urea, 5 mM CaCl_2_, 10 µM hemin, 0.7 mM oxidized glutathione, 0.1 mM dithiothreitol, and 0.2 mg/mL of protein, in buffer 40 mM Tris–HCl, pH 9.5 [59]. The active enzymes were purified using a Resource-Q column (GE-Healthcare, USA) with 0–400 mM NaCl salt gradient and 2 mL/min flow in 10 mM sodium acetate, pH 5.5, containing 1 mM of CaCl_2_.

### Steady-state kinetics

Five different substrates were selected for the kinetic characterization of the resurrected peroxidases: (i) Mn^2+^, which is oxidized to Mn^3+^ (Mn^3+^-tartrate complex ε_238_ 6500 M^−1^ cm^−1^); (ii) VA, whose oxidation product is veratraldehyde (ε_310_ 9300 M^−1^ cm^−1^); (iii) DMP, which dimerizes to coerulignone (ε_469_ 55,000 M^−1^·cm^−1^); (iv) ABTS, which is oxidized yielding the cation radical (ε_436_ 29,300 M^−1^ cm^−1^); and (v) RB5, whose disappearance/discoloration after oxidation was measured (ε_598_ 30,000 M^−1^ cm^−1^). These reactions were analyzed using a Thermo Scientific Biomate5 spectrophotometer, at 25 °C and the optimal pH and H_2_O_2_ concentration for each enzyme, determined using 50 mM Britton–Robinson (B&R) buffer (pH 2–10) and 2.5 mM ABTS as substrate (see Table 1 footnote). The sigmoid kinetic curves obtained for oxidation of DMP and ABTS enabled calculation of two sets of constants for AVP, corresponding to low and high efficiency oxidation sites, as reported for extant VP [40].

### pH and temperature stability

To study the effect of pH on enzyme stability, the resurrected peroxidases and extant LiPH8 were incubated in B&R buffer, pH 2–10, at 25 °C for 4 h. Then, the residual activity was estimated by the oxidation of ABTS (2.5 mM), under the conditions described above. For every enzyme, the activity after 1 min incubation at 25 °C in pH 5 buffer was taken as 100%, and the percentages of residual activity at the different pH conditions were referred to this value.

To study their thermal stability, the enzymes were incubated in 10 mM acetate, pH 5.5, for 10 min at 5 °C intervals in the range 25–85 °C. Residual activity was measured and calculated as described above. Temperature stability was presented as the 10 min *T*_50_, i.e., the temperature at which 50% of the activity was lost after 10 min incubation.

The effect of temperature on circular dichroism spectra was addressed studying the changes at 222 nm from 20 to 95 °C, 30 °C/h, using a Jasco J-815 spectropolarimeter equipped with a Peltier temperature controller and a thermostated cell holder on a 0.1 cm path length quartz cell. A final concentration of 6 µM pure enzyme in 10 mM acetate, pH 5.5 was used. *T*_m_ represents the temperature at the midpoint of the unfolding transition in the thermal melting profiles.

The effect of Mn^2+^ in the enzyme thermal inactivation and structure unfolding was analyzed by adding 1 mM SO_4_Mn to the incubation mixture, and measuring the residual activity by oxidation of 6 mM Mn^2+^, as described above.

## Additional files

**Additional file 1: Figure S1.** This figure includes three reconstructed sequences for each of the four nodes in the evolutionary pathway. **Figure S2.** provides the posterior probabilities for each amino acid in the 12 reconstructed sequences included in the previous figure. **Figure S3.** shows the proximal histidine environment in the ancestral sequences, compared with C*c*P and LiPH8. **Figure S4.** represents the environment of the catalytic tryptophan and homologous region in three ancestral peroxidases and LiPH8 as electrostatic surfaces.

**Additional file 2.** This file provides 113 sequences of Polyporales peroxidases from ten sequenced genomes in fasta format, aligned in a multiple fas (txt) file.

## Abbreviations

ABTS: 2,2′-azinobis (3-ethylbenzothiazoline-6-sulfonate)
ALiP: ancestral LiP
AVP: ancestral VP
CaCD: common ancestor of cluster-D peroxidases
CaPo: common ancestor of Polyporales peroxidases
C*c*P: cytochrome *c* peroxidase
DMP: 2,6-dimethoxyphenol
GP: generic peroxidase
*k*_cat_: catalytic constant
*K*_m_: Michaelis constant
LiP: lignin peroxidase
LiPH8: isoenzyme H8 of *P. chrysosporium* LiP
MnP: manganese peroxidase
RB5: Reactive Black 5
VA: veratryl alcohol
VP: versatile peroxidase
