# Supplementary material for: Experimental recreation of the evolution of lignin-degrading enzymes from the Jurassic to date
Source: Biotechnol Biofuels. 2017 Mar 16;10:67. doi: 10.1186/s13068-017-0744-x (PMC5356311; doi:10.1186/s13068-017-0744-x)
Supplement: Supplementary file 1 — Additional file 1: Figure S1. This figure includes three reconstructed sequences for each of the four nodes in the evolutionary pathway. Figure S2. provides the posterior probabilities for each amino acid in the 12 reconstructed sequences included in the previous figure. Figure S3. shows the proximal histidine environment in the ancestral sequences, compared with CcP and LiPH8. Figure S4. represents the environment of the catalytic tryptophan and homologous region in three ancestral peroxidases and LiPH8 as electrostatic surfaces. [file 13068_2017_744_MOESM1_ESM.pdf]

ADDITIONAL FILE 1: Supplemental Figures S1-S4

A

```
CaPo      VTCSDGVSTASNAACCAWFAVLDDIQANLFDGGQCGEEAHESLRLTFHDAIGFSPALAAQGKFGGGGADGSIITFADIETNFHANNGLDDIVDALKPFAD 100
CaPo-bis  VTCSDGVSTATNAACCAWFAVLDDIQANLFDGGQCGEEAREALRLTFHDAIGFSPALAAQGKFTGGGADGSIITFADIETSFHANNGLDDIVEALKHFAD 100
CaPo-tris ATCSDGIRTATNAACCAWFAVLDDIQANLFDGGQCGDQAREALRLTFHDAIGRSPALARQGKFTGGGADGSIITFADIELSYHANNGLEEIVEAQKPFAD 100
          .*****: **:*************************::*:*:********** ***** **** *************** .:******::**:* * ***
CaPo      KHNVSYGDFIQFAGAVGVSNCPGAPRLEFLAGRPNATAPSPDGLVPEPSDSVDKILARMADAGGFSPDEVVALLASHSVAAQDHVDPTIPGTPFDSTPST 200
CaPo-bis  KHNVSYGDFIQFAGAVGVSNCPGAPRLEFHAGRPNATAPSPDGLVPEPSDSVDKILARMSDAGGFTPDETVALLASHSVAAQRHVDPTIPGTPFDSTPGT 200
CaPo-tris KHNVSFGDFIQFAGAVGLSNCPGAPRLEFHAGRPNAIAPSPDGLVPEPSDSVDKILARMSDAGGFTPDDTVALLASHSVAAQDTVDPTIPGTPFDSTPGT 200
          *****:***********:*********** ****** **********************:*****:**:.************  **************.*
CaPo      FDTQFFLETLLKGTAFPGTGANSGEVKSPLKGEFRLQSDAAIARDPRTACEWQSFVNNQELMQSSFRAAMAKLANLGHDRSDLIDCSEVIPVPKPLAASA 300
CaPo-bis  FDTQFFVETLLKGTAFPGKGANSGEVKSPSPGEFRLASDAAIARDPRTACEWQSFVNNQEKMQSSFRAAMLKLAIQGHDRSDLIDCSEVIPVPKPLASSA 300
CaPo-tris FDTQFFVETLLKGTAFPGTGANTGEVKSPLKGEFRLASDAAIARHPRTACEWQSFVNNQEKMQASFRAAMAKLANQGHDRSDLVDCSEVIPVPKPLAASA 300
          ******:***********.***:******  ***** *******.*************** **:****** *** *******:*************:**
CaPo      TFPAGKTRSDIEQSCRSTPFPTLPTDPGPATSIPPV 336
CaPo-bis  TFPAGKTRKDIEQSCPSTPFPTLPTDPGPETSVPPV 336
CaPo-tris TFPAGKSRKDIEQSCPSTPFPTLPSDPGPATSIPPV 336
          ******:*.****** ********:**** **:***
```

B

```
CaCD      VTCPDGVNTATNAACCALFAVLDDIQENLFDGGECGEEAHESLRLTFHDAIGFSPALARQGKFGGGGADGSIITFSDIETNFHANGGIDEIVEVQKPFVA 100
CaCD-bis  VTCPDGVNTATNAACCALFAVVDDIQENLFDGGECGEEVHESLRLTFHDAIGFSPALARQGKFGGGGADGSIITFSDIETNFHANGGIDEIVEVQKPFVA 100
CaCD-tris VTCPDGVNTATNAACCALFAVVDDIQENLFDGGECGEEAHESLRLTFHDAIGFSPALARQGKFGGGGADGSIITFSDIETNFHANGGIDDIVEVQKPFVA 100
          *********************:****************.**************************************************:**********
CaCD      KHNMTAGDFIQFAGAVGVSNCPGAPRLEFLLGRPAATAPSPDGLVPEPFDSVDKILARFADAGGFSPDEVVALLASHSVAAADHVDPTIPGTPFDSTPST 200
CaCD-bis  KHNMTAGDFIQFAGAVGVSNCPGAPRLEFLLGRPAATAPSPDGLVPEPFDSVDKILARFADAGGFSPDEVVALLASHSVAAADHVDPTIPGTPFDSTPST 200
CaCD-tris KHNMTAGDFIQFAGAVGVSNCPGAPRLEFLLGRPAATAPSPDGLVPEPFDSVDKILARFADAGGFSPDEVVALLASHSVAAADHVDPTIPGTPFDSTPST 200
          ****************************************************************************************************
CaCD      FDTQFFVEVLLRGTLFPGTGGNQGEVKSALRGEIRLQSDHEVARDPRTACEWQSFVNNQAKMQKSFRAAMAKLAILGHDRSDLIDCSEVIPVPPPLAATA 300
CaCD-bis  FDTQFFVEVLLRGTLFPGTGGNQGEVKSALPGEIRLQSDHEVARDPRTACEWQSFVNNQAKMQKSFRAAMAKLAILGHDRSDLVDCSEVIPVPPPLAATA 300
CaCD-tris FDTQFFVEVLLRGTLFPGTGGNQGEVKSPLRGEIRLQSDNEVARDPRTACEWQSFVNNQAKMQKSFRAAMAKLAILGHDRSDLIDCSEVIPVPPPLAATA 300
          **************************** * ********.*******************************************:****************
CaCD      HFPAGLTRKDIEQSCRSTPFPTLSTDPGPATSVPPV 336
CaCD-bis  HFPAGLTRKDIEQSCRSTPFPTLSTDPGPATSVPPV 336
CaCD-tris HFPAGLTRKDIEQSCRSTPFPTLSTDPGPATSVAPV 336
          ********************************* **
```

C

```
AVP       VACPDGVNTATNAACCALFAVRDDIQQNLFDGGECGEEVHESLRLTFHDAIAFSPALEAQGQFGGGGADGSIAIFEDIETNFHANLGLDEIVNEQKPFIA 100
AVP-bis   VACPDGVNTATNAACCALFAVRDDIQQNLFDGGECGEEVHESLRLTFHDAIAFSPALEAQGQFGGGGADGSIAIFEDIETNFHANLGLDEIVNEQKPFIA 100
AVP-tris  VACPDGVNTATNAACCALFAVRDDIQQNLFDGGECGEEVHESLRLTFHDAIAFSPALEAQGQFGGGGADGSIAIFEDIETNFHANLGLDEIVNEQKPFIA 100
          ****************************************************************************************************
AVP       RHNMTTADFIQFAGAVGVSNCPGAPQLDFFLGRPDATQPAPDGLVPEPFDTVDQILARMADAGGFDPIETVWLLTSHTIAAADHVDPTIPGTPFDSTPEL 200
AVP-bis   RHNMTTADFIQFAGAVGVSNCPGAPQLDFFLGRPDATQPAPDGLVPEPFDTVDQILARMADAGGFDPIETVWLLTSHTVAAADHVDPTIPGTPFDSTPEL 200
AVP-tris  RHNMTTADFIQFAGAVGVSNCPGAPQLDFFLGRPDATQPAPDGLVPEPFDTVDQILARMADAGGFDPIETVWLLASHTIAAADHVDPTIPGTPFDSTPEL 200
          **************************************************************************:***:*********************
AVP       FDTQFFIETQLRGTLFPGTGGNQGEVESPLRGEIRLQSDHLLARDSRTACEWQSFVNNQPKLQKSFQAAFHDLSMLGHDVNDLIDCSEVIPIPPPPTSTA 300
AVP-bis   FDTQFFIETQLRGTLFPGTGGNQGEVESPLRGEIRLQSDHLLARDSRTACEWQSFVNNQPKLQKNFQAAFHDLSVLGHDVNDLIDCSEVIPIPPPPASTA 300
AVP-tris  FDTQFFIETQLRGTLFPGTGGNQGEVESPLRGEIRLQSDHLLARDSRTACEWQSFVNNQPKLQKNFQAAFHDLSMLGHDVNDLIDCSEVIPIPPPPTSTA 300
          ****************************************************************.*********:*********************:***
AVP       HFPAGLTNADVEQACAETPFPTLPTDPGPATSVAPV 336
AVP-bis   HFPAGLTNADVEQACAETPFPTLPTDPGPATSVAPV 336
AVP-tris  HFPAGLTNADVEQACAETPFPTLPTDPGPATSVAPV 336
          ************************************
```

D

```
ALiP      VACPDGVNTATNAACCALFAVRDDIQQNLFNGGQCGDEAHESLRLTFHDAIAFSPALEAQGQFGGGGADGSIVIFSDIETNFHANIGLDEIVAIQKPFIA 100
ALiP-bis  VACPDGVNTATNAACCALFAVRDDIQQNLFNGGQCGDEAHESLRLTFHDAIAFSPALEAQGQFGGGGADGSIVIFSDIETNFHANIGLDEIVAIQKPFIA 100
ALiP-tris VACPDGVNTATNAACCALFAVRDDIQQNLFNGGQCGDEAHESLRLTFHDAIAFSPALEAQGQFGGGGADGSIVIFSDIETNFHANIGLDEIVAIQKPFIA 100
          ****************************************************************************************************
ALiP      RHNMTVADFIQFAGAVGVSNCPGAPQLNFFLGRPDATQPAPDGLVPEPFDTVDQILARMADAGEFDELETVWLLIAHTVAAANDVDPTIPGTPFDSTPEL 200
ALiP-bis  RHNMTVADFIQFAGAVGVSNCPGAPQLNFFLGRPDATQPAPDGLVPEPFDTVDQILARMADAGEFDELETVWLLIAHTVAAANDVDPTIPRTPFDSTPEL 200
ALiP-tris RHNMTVADFIQFAGAVGVSNCPGAPQLNFFLGRPDATQPAPDGLVPEPFDTVDQILARMADAGEFDELETVWLLIAHTVAAANDVDPTIPRTPFDSTPEL 200
          ****************************************************************************************** *********
ALiP      FDSQFFIETQLRGTLFPGTGGNQGEVESPLKGEMRLQSDHLLARDSRTACEWQSFVNNQPKLQKNFQFVFEALSMLGQDPNDLIDCSEVIPIPPPLTLTP 300
ALiP-bis  FDSQFFIETQLRGTAFPGTGGNQGEVESPLKGEMRLQSDHLLARDSRTACEWQSFVNNQPKLQKNFQFIFEALSMLGQDPNNLIDCSEVIPIPPPLTLTP 300
ALiP-tris FDSQFFIETQLRGTLFPGTGGNQGEVESPLKGEIRLQSDHLLARDSRTACEWQSFVNNQPKLQKNFQFVFEALSMLGQDPNDLIDCSEVIPIPPPLTLTP 300
          ************** ******************:**********************************:************:******************
ALiP      AASHFPAGKTNKDVEQACAETPFPTLPTDPGPATSVAPVPPSPAA 345
ALiP-bis  AFSHFPAGKTNADVEQACAETPFPTLPTDPGPATSVAPVPPSPAA 345
ALiP-tris GAAHFPAGKTNKDVEQACAETPFPTLPTDPGPATSVAPVPPSPAA 345
          . :******** *********************************
```

Figure S1. Twelve reconstructed sequences representative of the different nodes. Most probable and alternative (bis/tris) sequences (mature proteins) for CaPo (**A**, 88-92% identity), CaCD (**B**, 98-99%), AVP (**C**, 99%) and ALiP (**D**, 98-99%) nodes were obtained by Monte Carlo sampling (yielding 5000 sequences/node using 0.5 threshold) followed by random sampling (see Methods for sampling strategy) yielding 3 sequences/node (red and blue, typical Mn-binding site and catalytic tryptophan, respectively; other colors, see **Fig. 2**).

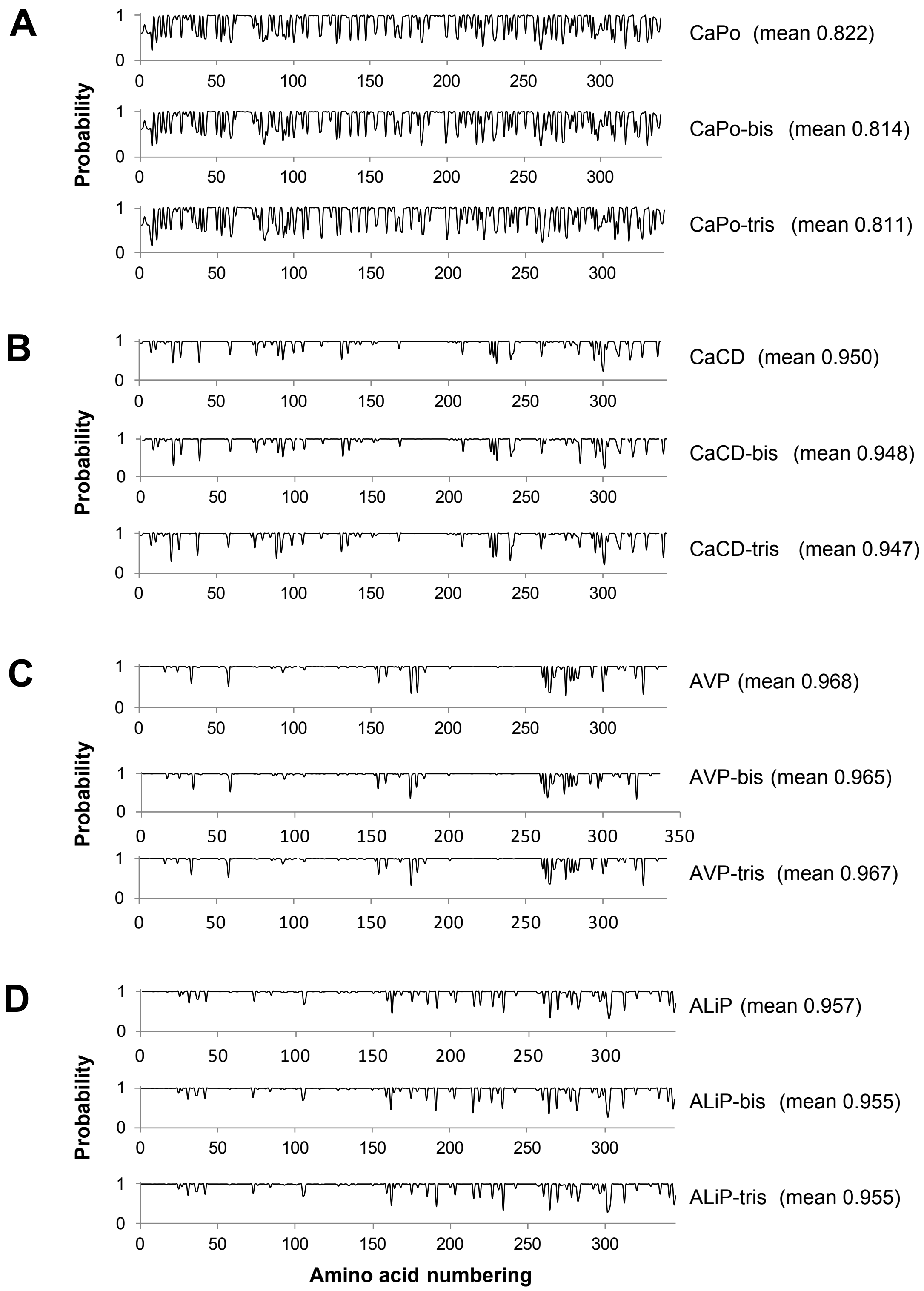

Figure S2. Posterior probability for each amino acid of the 12 reconstructed ancestral sequences shown in **Fig. S1**. The most probable sequence for nodes CaPo (**A**), CaCD (**B**), AVP (**C**) and ALiP (**D**), and two alternative sequences (bis and tris) for each of them (see Methods for sampling strategy) are included. The probabilities of Trp172 in AVP and ALiP sequences are 0.999 and 1.000, respectively; and that of Asn183 in ALiP sequences is 0.993. The mean probabilities for every reconstructed sequence are also shown.

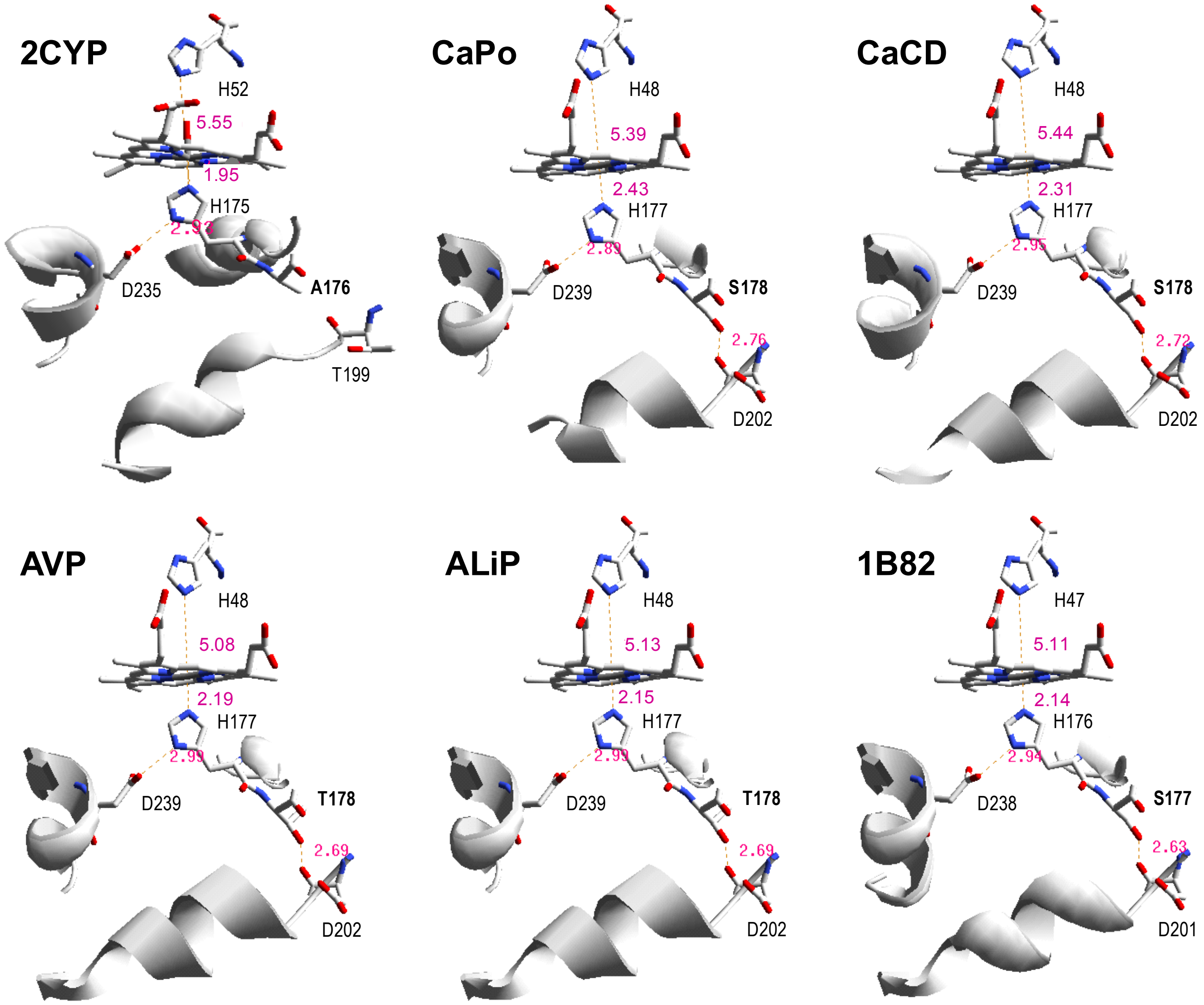

Figure S3. Proximal histidine, neighbor residues and distances (Å) in ancestral peroxidases (His177) and extant C*c*P (2CYP; His175) and *P. chrysosporium* LiPH8 (1B82; His176). His177-Asp239 and Ser/Thr178-Asp202 H-bonds would affect the position of the proximal histidine in the ancestral peroxidases, and consequently the heme iron electron-deficiency (homologous H-bonds are present in LiPH8, but the second one is absent from C*c*P). Distal histidine (His48 in the ancestors, His52 in CcP, and His47 in LiPH8) is also shown.

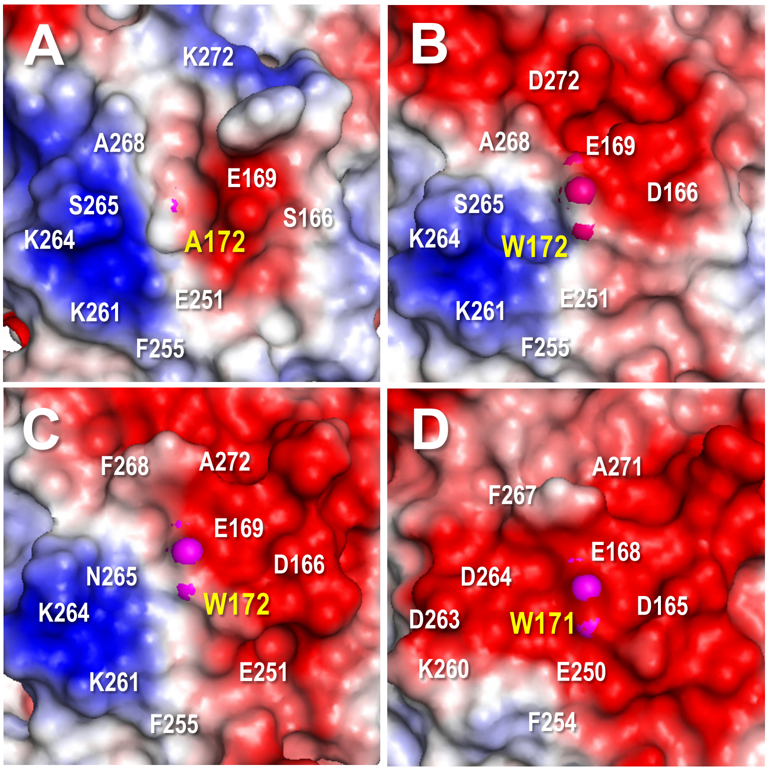

Figure S4. Surface environment of catalytic tryptophan. Electrostatic surfaces computed for ancestral CaCD (**A**), AVP (**B**) and ALiP (**C**) homology models, and extant LiPH8 (**D**) crystal structure (PDB 1B82) showing the environment of the exposed tryptophan (magenta spheres, yellow labels) in **B**-**D**, and the equivalent alanine residue in **A** (the positions of neighbor residues are also indicated). The presence of the catalytic tryptophan in AVP and ALiP and the absence of a net negative environment, as found in LiPH8 that is unable to oxidize RB5, contribute to the oxidation of this anionic dye by the two ancestral enzymes. The opposite effect is expected for VA, whose cation radical would be stabilized by the negative environment in LiPH8.
